# Low-Cost Chestnut-Based Biocarbons Physically Activated via CO_2_ or Steam: Evaluation of the Structural and Adsorption Properties

**DOI:** 10.3390/ma18071497

**Published:** 2025-03-27

**Authors:** Barbara Charmas, Barbara Wawrzaszek, Katarzyna Jedynak, Agata Jawtoszuk

**Affiliations:** 1 Department of Chromatography, Institute of Chemical Sciences, Faculty of Chemistry, Maria Curie-Sklodowska University, Maria Curie-Sklodowska Sq. 3, 20-031 Lublin, Poland; barbara.charmas@mail.umcs.pl (B.C.); ajawtoszuk111@wp.pl (A.J.); 2 Institute of Chemistry, Faculty of Natural Sciences, Jan Kochanowski University, Uniwersytecka Str. 7, 25-406 Kielce, Poland; katarzyna.jedynak@ujk.edu.pl

**Keywords:** biomass conversion, activated biocarbon, physical activation, porous structure, surface chemistry, adsorption of pollutants

## Abstract

The aim of this paper was to obtain activated biocarbons from the natural biomass of horse chestnut seeds (*Aesculus hippocastanum*) by physical activation with two different activating agents, carbon dioxide and water vapor, and to evaluate their structural and adsorption properties. The effect of the pyrolysis atmosphere on the surface development and porosity as well as the structure and adsorption properties of the materials in relation to the selected organic adsorbates (tetracycline (TC), naproxen (NPX), and methyl orange (MO)), which may constitute a potential contamination of the aquatic environment, was evaluated. Activated biocarbons were characterized using N_2_ low-temperature adsorption/desorption, Raman and FT-IR spectroscopy, and thermogravimetric analysis (TGA). The nature of the surface (pH_pzc_ and Boehm titration) was also studied. Micro/mesoporous biocarbons were obtained with an S_BET_ area in the range of ~534 to 646 m^2^/g, in which micropores constituted ~70%. It was proved that the obtained materials are characterized by high adsorption values (~120 mg/g, ~150 mg/g, and ~252 mg/g) and removal rates %R (~80%, ~95%, and ~75%) for TC, NPX, and MO, respectively. The results indicate that chestnut-derived activated biocarbons are a promising, cost-effective and environmentally friendly alternative for removing organic contaminants from aqueous solutions. Future research should focus on optimizing activation parameters and assessing the long-term performance of adsorbents.

## 1. Introduction

Over the past decades, the world has been witnessing an intense population growth and constant development of industry. The consequence of these changes is an increase in the amount of household and industrial waste [1,2]. A significant group of wastes are toxic organic substances, which get into the aquatic environment in huge quantities. Organic contaminants, including dyes, medicines, pesticides, etc., are difficult to biodegrade and pose a threat to aquatic ecosystems as well as human health and life [3,4]. This issue is a huge challenge for the modern world of science [5,6]. Therefore, a search for strategies to eliminate these pollutants is underway. There are many effective methods of removing organic substances from the aqueous environment, such as flocculation, filtration and chemical precipitation [7,8], advanced oxidation processes [9,10], biological oxidation [11], or adsorption, among others [12,13,14]. However, the most effective method of purification seems to be adsorption [15,16,17]. The method is effective, efficient, and cheap as well as being environmentally friendly, and most importantly it does not cause secondary pollution. Contaminant removal efficiency varies depending on the type of contamination and the type of adsorbent. The conventional adsorbents, e.g., ion exchange resins or commercial activated carbons, are expensive and not ecological, and so new potential carbon precursors are sought from which adsorbents are finally obtained.

A significant challenge for scientists is the use or elimination of waste, namely biomass [1,18,19]. The source of waste biomass can be municipal and green waste, waste from the agricultural industry as well as forestry, agricultural waste (straw, stalks, leaves, seeds), waste from the agri-food industry, and many others. It should be noted that the composition and structure of biomass vary greatly depending on the origin, plant species, processing and storage methods, etc. Therefore, extensive research into the possibility of using different types of biomass is extremely important because various materials will undergo transformations in different ways, resulting in products of great diversity. Significant amounts of forest and agricultural residues are not recycled, often remaining unused and are consequently wasted. The natural decomposition of biomass releases CO_2_ and other greenhouse gases into the atmosphere, increasing anthropogenic environmental pollution. A partial solution to the problem of the huge amount of residual organic biomass can be to process it into biocarbon. Currently, intensive research is being carried out on the production and possibility of using biocarbons due to their potential for carbon sequestration, soil quality improvement [20,21], climate change mitigation, catalysis, wastewater treatment [22,23], energy storage [24], and waste management.

The attempts to use biomass to obtain effective biocarbon adsorbents have yielded excellent results. As a result of thermal transformations (pyrolysis), biocarbons are obtained with a huge diversity in structure, nature, and surface chemistry, giving great opportunities for the use of these materials, especially in adsorption processes. The desired properties depend on the characteristics of the starting material, but they can also be controlled by selecting the appropriate biomass, activator, and activation method. Biocarbons derived from biomass are an excellent alternative to the materials obtained from non-renewable sources. Activated biocarbons are used in the processes of removing organic and inorganic water pollutants due to their good adsorption properties as well as their environmentally friendly properties, their great efficiency, and their much lower energy consumption during production and in their elimination of secondary environmental pollutants.

It should be noted that the properties of raw biocarbon mean it is not suitable for use in the adsorption processes of pollutants from water and air. For this purpose, carbon adsorbents must have a developed surface area and porosity, as well as a wide range of functionalities. Such properties are created in the activation process. Selecting the optimal activating agent is not an easy task, and it is necessary to strive to minimize any secondary contamination and improve its adsorption properties. Various methods are used to activate carbon materials, including physical [25] or chemical activation [26,27]. Although chemical activation (using NaOH, KOH, ZnCl_2_, and others) allows for better surface development, the removal of residual activating agents after the activation process requires significant amounts of water and causes secondary environmental pollution. Physical activation (CO_2_, H_2_O, air, or air/H_2_O mixture) is more in line with the principles of green chemistry. The most commonly used physical activators are CO_2_ and over-heated steam [28,29]. The course of their reaction with carbon is endothermic and this facilitates the control of the activation process. CO_2_ is the preferred activator because it is less reactive at high-temperatures and its use promotes the formation of micropores [1,30,31,32]. On the other hand, activation with water vapor causes the micropores to widen and so biocarbons are characterized by a smaller volume of micropores at the expense of increasing the volume of meso- and macropores. Importantly, the addition of oxygen (in the form of CO_2_ or H_2_O) allows its simultaneous chemisorption on the carbon surface which results in the formation of surface functional groups [33].

The aim of this research is to analyze the possibility of horse chestnut seed conversion into high-quality activated biocarbons that can be used in the processes of adsorption purification of organic pollutants from water. Despite numerous studies on the possibility of converting chestnut biomass into biocarbons, the SCOPUS database (in the years 2018–2024, data from 11 March 2025) indicates the publication of 51 papers on the use of chestnuts in biocarbon production (keywords: chestnut, pyrolysis). Only 29 papers deal with the use of chestnut seeds or shells. To the best of our knowledge, only two papers are about the use of horse chestnut seeds [34,35]. Horse chestnut (*Aesculus hippocastanum*) seeds are readily available, being abundant in many areas of Europe and other parts of the world, especially in cities and green areas. The use of seeds does not require cutting down trees, which is important from the point of view of sustainable development. In addition, seeds have a different structure and chemical composition from that of wood or shells, so they constitute a new type of biomass. With this in mind, the chestnut seeds were used as the initial biomass. Biocarbons were obtained using different pyrolysis parameters and activators (CO_2_, H_2_O). The structural, thermal, and adsorption characteristics of biocarbons were determined using low-temperature N_2_ adsorption, TGA, FTIR, and Boehm titration. The adsorption studies of organic molecules, such as an antibiotic (tetracycline, TC), a nonsteroidal anti-inflammatory drug (naproxen, NPX), and a dye (methyl orange, MO), were carried out to determine the total adsorption capacities of the materials. A thorough characterization combined with the determination of total sorption capacities will allow us to assess the applicability of the obtained biocarbons in the environmental purification processes.

## 2. Materials and Methods

### 2.1. Biocarbons Preparation

The starting material is an organic biomass with a large carbon content and a low ash content. These are inedible, large seeds commonly found in the immediate vicinity (Lublin, Poland). The crushed and dried (105 °C, 48 h) raw material characterized by various shapes and sizes of grains was placed in a quartz reactor and free pyrolysis was carried out with a temperature increase of 10 °C/min. The influence of time, pyrolysis atmosphere, and the activating agent on the properties of the obtained biocarbons was investigated. Biocarbons were determined according to the conditions and duration of the process: the samples of (1) 800-CO_2_-1 and (2) 800-CO_2_-3 were pyrolyzed and activated in a CO_2_ atmosphere and heated at the maximum temperature for 1 and 3 h, respectively (the whole process was carried out in CO_2_); (3) 800-mix-1 was obtained by pyrolysis in a N_2_ atmosphere (10 °C/min to 800 °C), and then the material was heated in CO_2_ for 1 h and cooled to room temperature in N_2_; (4) 800-H_2_O-1 was obtained by pyrolysis in N_2_ (10 °C/min to 800 °C), then subjected to superheated steam (Peristaltic pump, Cole Palmer, USA, the flow rate 0.6 mL/min) for 1 h, and then cooled to room temperature in N_2_. The inert gas (N_2_) and CO_2_ flows were 160 cm^3^/min and 120 cm^3^/min, respectively. The obtained biocarbons were fractionated and the 0.4–0.63 mm fraction was selected for further research.

### 2.2. Methods

The estimation of textural characteristics was based on the low-temperature (−196 °C, ASAP 2405N Analyzer, Micromeritics, Norcross, GA, USA) N_2_ adsorption–desorption isotherms data. The specific surface area (S_BET_) was calculated according to the standard BET method [36]. The total pore volume (V_p_) was determined from the nitrogen adsorption at p/p_0_ = 0.98–0.99 (p and p_0_ are the N_2_ equilibrium and saturation pressures, respectively). The nitrogen desorption data were used to calculate the pore size distributions (PSD, f_v_(R_p_)~dV_p_/dR_p_) using a regularization procedure under a non-negativity condition (f_V_(R_p_) ≥ 0 for any R_p_) with a fixed parameter of regularization α = 0.01. The slit-shaped pores model was used. The f_V_(R_p_) functions were used to calculate the contribution of micropores (S_micro_ and V_micro_ at a pore radius R_p_ < 1 nm), mesopores (S_meso_ and V_meso_ at a pore radius 1 nm ≤ R_p_ ≤ 25 nm), and macropores (S_macro_ and V_macro_ at a pore radius R_p_ > 25 nm). The procedure of PSD calculation is given in [37,38].

The total pore volume (V_total_) of biocarbons was determined by filling the free intergranular spaces with methanol (CH_3_OH, analytical reagent grade, Standard, Lublin, Poland). Methanol was added to the dried biocarbon weights (~0.2 g) until the material became cohesive and compact. V_total_ was determined based on Equation (1), which is as follows:

V_total_ = V_MeOH_/m_s_ (1)

where V_total_ is the total pore volume [cm^3^/g], V_MeOH_ is the volume of methanol used to fill pores and intergranular spaces [cm^3^], and m_s_ is the sample mass [g].

For each biocarbon, the analyses were repeated 5 times.

The bulk density was determined by analyzing the volume of a specific mass of biocarbon using Equation (2), which is as follows:

ρ = m_s_/V_s_ (2)

where ρ is the bulk density [g/cm^3^], m_s_ is the sample mass [g], and V_s_ is the sample volume [cm^3^].

For each biocarbon, the analyses were repeated 5 times.

The number of surface acid and basic functional groups was determined by potentiometric titration (716 DMS Titrino, Metrohm, Herisau, Switzerland) using the method proposed by Boehm [39,40]. The method is based on the stoichiometric reaction of the ion exchange of H^+^ ions with Na^+^ ions derived from the base. The samples (~0.2 g) were poured with 10 mL of a HCl (determination of basic functionalities, 0.05 N) or NaOH (determination of acidic functionalities, 0.05 N) solution and placed in a shaker (25 °C, 24 h). The hydrochloric acid (HCl—35%) and sodium hydroxide (NaOH) purchased from Standard (Poland) were of analytical reagent grade. Next, the suspensions were filtered with a syringe filter and titrated with the NaOH solution, respectively, with the total content of acid groups determined based on back-titration [41]. For each material, the analyses were repeated 3 times.

Thermogravimetric analysis was conducted using a Derivatograph C (Paulik, Paulik, and Erdey, MOM, Budapest, Hungary). The ~10 mg samples were placed in corundum crucibles and Al_2_O_3_ was the reference substance. Measurements were made in the air and in a nitrogen atmosphere (20–1000 °C, 10 °C/min). The TG and DTA curves were recorded. The data of the experiments conducted in the air atmosphere were used to determine the thermal stability of the biocarbons. Moreover, a proximate analysis was made. The volatile matter content (VM%) was estimated from the TGA data (N_2_, 150–900 °C). The ash content (A%) was found to be a residue after the complete thermal degradation of organic substances in the O_2_ atmosphere. The fixed carbon content (FC%) was estimated from the relationship FC% = 100 − (A% + VM%). The parameters were determined referring to the dry matter. The thermostability index was estimated based on the following formula: C_thermo_ = %FC/(%FC + %VC) [42]. For better characterization of carbon contained in the biomaterials, the TG was corrected in accordance with the assumptions presented by Harvey et al. [43] and the recalcitrance index was determined.

Reflex Raman spectroscopy (Microscope DMLM Leica Research Grade, Reflex, Renishaw, UK) with an argon laser of 785 nm wavelength was used to determine the degree of the biocarbon structure ordering.

The FT-IR spectra (Perkin-Elmer Spectrum 400-FT-IR/FT-NIR, Perkin-Elmer, Waltham, MA, USA) were recorded in the range of 4000–650 cm^−1^ with a 4 cm^−1^ resolution.

Surface pH was determined by pH measurements (716 DMS Titrino, Metrohm, Switzerland) of aqueous solutions after 24 h of contact with a biocarbon (0.4 g biocarbon, 20 mL distilled water, shaking 140 rpm. for 24 h, 25 °C) [44]. The tests were repeated 3 times.

The total adsorption capacity measurements of the biocarbons were made for the selected pollutants: tetracycline hydrochloride (TC, purity ≥ 95%, Glentham, UK), naproxen sodium (NPX, purity ≥ 98%, Glentham, UK), and methyl orange (MO, Chempur, Lublin, Poland). The measurements were performed using different initial concentrations: 50 mg/L and 300 mg/L (for TC and NPX, respectively), as well as 700 mg/L (MO). The preliminary studies of adsorption kinetics allowed us to assess the time needed to establish the adsorption equilibrium in the studied adsorbent/adsorbate systems. The samples (20 mg) were put into contact with 10 mL of impurities solution. The suspensions were shaken in the thermostat (140 rpm, 25 °C, 120 h). The equilibrium was controlled after 12, 24, 48, 72, 96, and 120 h. The equilibration times used for TC, NPX, and MO were 120 h, 12 h, and 24 h, respectively. The concentration of impurities was determined using spectrophotometric measurements (Spectrophotometer UV-VIS Thermo Spectronic Helios Gamma, Spectro-Lab, Warsaw, Poland) at the wavelengths appropriate for a specific contaminant, determined based on the spectra of the samples under study (TC: λ_max_ = 377 nm; NPX: λ_max_ = 230 nm; MO: λ_max_ = 464 nm). The procedure was repeated 3 times, and the data were presented as the average value ± standard deviation (SD).

The adsorption capacity q_e_ (mg/g) and the removal percentage (%R) of all impurities were calculated using Equations (3) and (4), respectively, which are as follows [45]:

q_e_ = V(C_0_ − C_e_)/m (3)

%R = [(C_0_ − C_e_)/C_0_] × 100 (4)

where C_0_ and C_e_ are the initial and equilibrium adsorbate concentrations, mg/L, m is the adsorbent mass in g, and V is the volume in L.

## 3. Results and Discussion

The selected precursor (chestnut seeds) is characterized by great structural heterogeneity because it contains both the outer skin and the pulp of the seeds. Due to the different efficiency of biomass activation as a result of the use of different activators, the pyrolysis yield (PY) was evaluated at the beginning. PY was determined on the basis of mass loss during the pyrolysis process (Table 1). The largest yield was found for the samples obtained as a result of 1 h pyrolysis in the presence of CO_2_ (800-CO_2_-1, 800-mix-1) because about 20% of the material remained in the form of biocarbon. Prolongation of the activation time (800-CO_2_-3) or the use of H_2_O as an activating agent (800-H_2_O-1) was associated with a very intense mass loss due to the thermal degradation of carbon structures.

**Figure 1:**
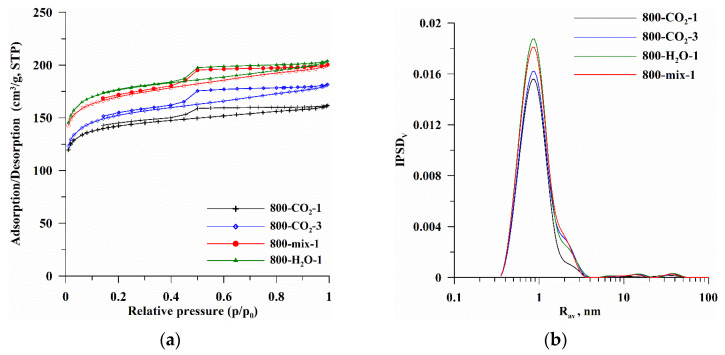
N_2_ adsorption/desorption isotherms (**a**) and pore volume distribution curves (**b**) determined for CO_2_ and H_2_O activated biocarbons.

**Figure 2:**
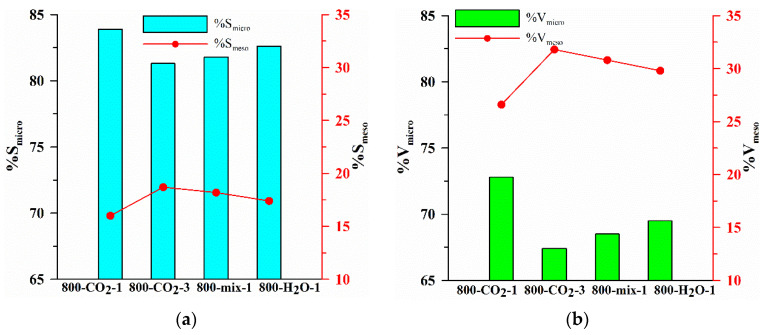
Changes in the surface (**a**) and volume (**b**) of the micro- and mesopores.

Figure 1 presents the isotherms of N_2_ adsorption/desorption (Figure 1a) and pore volume distributions (Figure 1b) determined for the studied biocarbons. The isotherm shape analysis (Figure 1a, type IV) indicates that micro/mesoporous materials with a significant share of micropores were obtained (Table 1, Figure 1 and Figure 2). The dominant pores radii of these biocarbons are below 1 nm (R_dom_, Table 1), while the average pores radii, due to the presence of mesopores, are ~1.3 nm (R_av_, Table 1). Different methods of modification resulted in an increase in the intensity of the IPSD_V_ bands (Figure 1b), indicating an increase in pore volume and the gradual formation of mesopores (Table 1, Figure 1b). Biocarbons exhibit complex hysteresis loop characteristics (H2, H4) because their structure is not homogeneous [46]. The shapes of the loops (Figure 1a) confirm the participation of bottle-shaped mesopores, characterized by a narrow entrance to a wide interior as well as slit-shaped pores formed between planes of different mutual inclinations. Such a shape of pores is characteristic of the materials obtained from biomass, and the small values of the “w” factor (Table 1) indicate a good fit of the assumed pore model [37,38].

The initial carbonizates obtained at 800 °C in the N_2_ atmosphere (S_BET_~0.1 m^2^/g) were characterized by an undeveloped surface and porous structure. This is due to the initial properties of the biomass, which was very compact, and during the thermal process in N_2_ there were not the right conditions for surface activation and pore formation. The use of activating factors enabled the intensive development and increase in the surface area and volume of micropores, as well as the formation of a certain number of mesopores (Table 1, Figure 1). Biocarbons activated in the CO_2_ atmosphere during the entire thermal process (800-CO_2_-1 and 800-CO_2_-3) have a specific surface area of 534.6 and 569.5 m^2^/g, for 800-CO_2_-1 and 800-CO_2_-3, respectively, of which more than 80% are micropores (Table 1, Figure 2). Extending the activation process to 3 h (sample 800-CO_2_-3) resulted in only a slight increase in S_BET_ with a simultaneous decrease in the micropores share (%S_micro_, %V_micro_) and an increase in the mesopores share (%S_meso_, %V_meso_, Figure 2). Activation generally results in the removal of weakly bound carbon structures. The result is an initial increase in the number of micropores and their size compared to carbonizates. In some places, the walls of the pores break and degrade, causing a decrease in microporosity and a gradual increase in mesoporosity (Figure 2a,b). At the same time, the prolongation of pyrolysis with simultaneous activation (sample 800-CO_2_-3) resulted in a significant decrease in pyrolysis efficiency compared to the 1 h process (Table 1), thus becoming uneconomical. More efficient pyrolysis was carried out under the conditions where the oxidizing gas (CO_2_) was used only in the activation step (800-mix-1). This is because CO_2_ used during the entire pyrolysis process (800-CO_2_-1 and 800-CO_2_-3) constantly oxidized the carbon material, causing its intensive thermal degradation, which increased the degree of thermal oxidative degradation and reduced the carbon content (800-CO_2_-1 and 800-CO_2_-3, Table 1). The use of CO_2_ only in the activation stage (800-mix-1) enabled the initial pyrolysis of the biomass and the subsequent activation of the biocarbon, resulting in an effective development of surface area and porosity without intensive mass losses of carbon.

The most developed surface was characterized by biocarbon activated by the superheated steam (800-H_2_O-1, Table 1). However, since the activation of H_2_O is more intense than the process carried out in CO_2_, a very low pyrolysis yield was observed as a result of the applied conditions (800-H_2_O-1, PY~9.6%, Table 1). A significant mass loss in the pyrolysis and activation stage increased the ash content of the material, fundamentally changing the chemical nature of the surface.

By filling the pores and intergranular spaces with methanol, the total volume of pores (micro, meso, and macro, as well as the voids between the particles, V_total_) was determined, which is about 1.1–2 cm^2^/g (Table 1). The largest V_total_ value for 800-mix-1 (1.976 cm^3^/g) is due to the preservation of the structure of particles in the pyrolysis stage and the significant development of porosity. On the other hand, the smallest value of V_total_ (for 800-H_2_O-1, 1.125 cm^3^/g) is associated with an intensive mass loss in the pyrolysis stage and a significant diminution in biocarbon particles. The increase in the bulk density of biocarbons (0.24 to 0.27 g/cm^3^, Table 1) was caused by a decrease in biocarbon particles due to activation.

**Figure 3:**
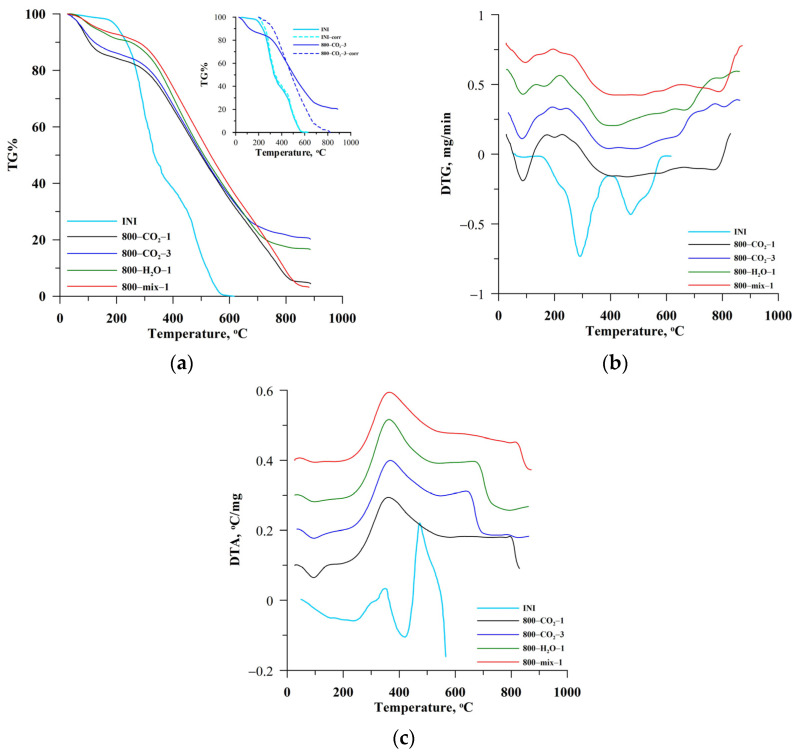
TG% (**a**), DTG (**b**), and DTA (**c**) curves as well as the comparison of uncorrected (solid lines) and corrected TG% thermograms (inset Figure 3a; dotted lines) for the biocarbons.

Figure 3a–c presents the results of thermal analysis (TG%, DTG, and DTA) obtained for the initial biomass (INI) and the biocarbons. Typically, thermograms obtained for the materials with a developed surface contain three main areas related to the mass loss. The first area, up to ~200 °C, is caused by the loss of moisture and unbound water from the surface and pores of the tested materials. The second area in the temperature range of ~200 °C to 550 °C for the initial biomass is related to the thermal decomposition of cellulose components and other aromatic structures. For the activated biocarbons, this area is shifted significantly towards higher temperatures, indicating that the biocarbons are better ordered and more thermally stable than the initial biomass. This process can take place in many stages, depending on the structure of the initial biomass and the nature of the carbon structures obtained in the pyrolysis process. As can be seen from the curves in Figure 3a, the method of activation affects also the thermal stability of biocarbons. The third area of mass loss is caused by the thermal decomposition of inorganic residues.

The shift in the temperature range of thermal degradation of biocarbons is visible on the TG% (Figure 3a), DTG (Figure 3b), and DTA (Figure 3c) curves. It has been shown that the temperature range of thermal transformations is shifted depending on the activation method. The initial biomass decomposes from ~200 °C due to the significant content of volatile, unbound carbon structures. The initial stage is the desorption of moisture and volatile compounds bound poorly to the lignocellulosic structures of the organic matter. The next stages of mass loss are related to the degradation of large organic molecules and their combustion in an oxygen atmosphere. The chestnut decomposition ended at ~570 °C (Figure 3). The course of the analyzed curves shows that the thermal degradation of biocarbons starts in the range of slightly higher temperatures (>300 °C). The most stable are the best-structured biocarbons 800-mix-1 and 800-CO_2_-1.

There are many ways to assess the thermostability of carbonaceous materials. In the presented study, two parameters assessing the stability of biocarbons were determined. The first one is the C_thermo_ parameter, indicating the mass share of the stable fraction in the biocarbon [40]. The second parameter, the recalcitrance index (R50), is based on the assumption that the amount of energy required to oxidize a given amount of biocarbon will depend on the environment and how the carbon atoms bind [43]. This indicates that a poorly ordered biocarbon with a higher proportion of C-C systems should have lower thermal stability than the structured biocarbons with C=C or aromatic structures. The recalcitrance index (R50) is determined based on the thermograms after correction for water and ash content (Figure 3a, inset, dotted lines) because it concerns the transformation of the carbon contained in the material but not the material as a whole. As can be seen (Figure 3a), these curves are in the range of 0–100% TG due to the analysis of the thermal degradation of only the carbon contained in the tested materials. The different course of the corrected curves confirms the different thermal stability of the materials, resulting from different activation conditions.

Thermal analysis of biocarbons was also performed in an inert atmosphere (N_2_). Such a procedure allows the study of mass losses resulting from biocarbon transformations under the influence of heating; however, without the oxidizing agent (air) there is no oxidative thermal degradation process, but only the desorption of weakly bound carbon compounds (%VC, volatile carbon) in the ordered carbon structures. The data in Table 2 show that the ash content (%A) in the materials varied and depended on the method of activation. The initial chestnuts contained only 1.93% ash, and the largest amount of ash was contained in the biocarbons 800-CO_2_-3 and 800-H_2_O-1 (23.5% and 18.33%, respectively). This is well correlated with the pyrolysis yield (PY, Table 1) because the largest mass loss was observed for these materials. The volatile compound content (%VC) was the largest for the initial material (INI, %VC = 75.9%, Table 2), but after pyrolysis and activation it was significantly reduced (from ~17% to ~26%). At the same time, an increase in the content of fixed carbon (%FC, 50–79.4%, Table 2) was observed in the form of thermally stable, ordered carbon structures. The thermal stability is also evidenced by the increasing values of the C_thermo_ and R50 parameters (Table 2). The most stable is 800-mix-1 biocarbon.

**Figure 4:**
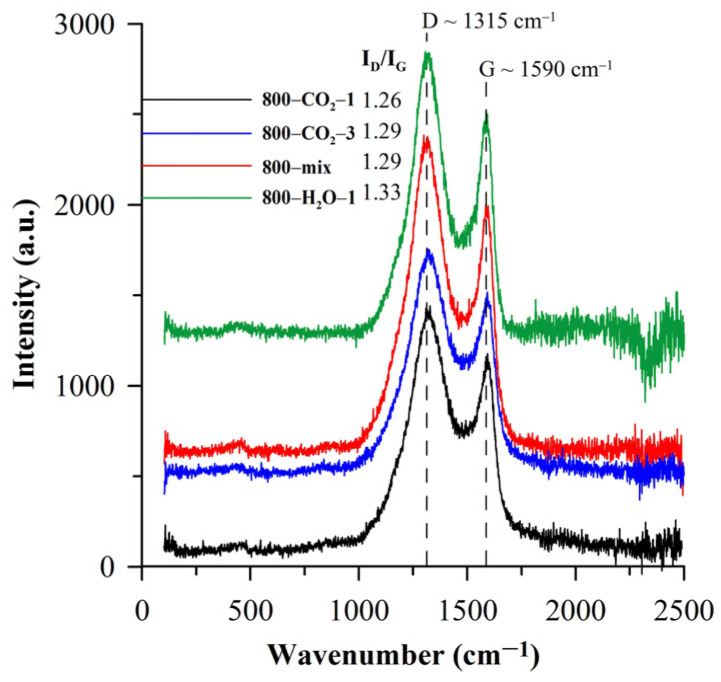
Raman spectra of the activated biocarbons.

The amorphous and nanocrystalline structure of biocarbons was proved based on the Raman spectra (Figure 4). Carbon materials are usually characterized by wide bands in the 1300 (D-band)–1600 cm^−1^ (G-band) range. D-band (~1315 cm^−1^, Figure 4) results from the presence of nanocrystalline carbon structures and indicates the vibration of breathing mode in the aromatic carbon rings. However, G-bands (~1590 cm^−1^) arise from sp^2^ carbon pairs stretching vibrations in both rings and chain structures. As follows from the presented spectra, the activation does not change the position of the bands but increases their intensity. The ratio of D- and G-band intensities (I_D_/I_G_, Figure 4) reflects the type of structure of carbon materials. Such a relationship allows for determining the content of the sp^3^ phase and the biocarbon prevailing structure [47]. The calculated I_D_/I_G_ ratios (Figure 4) increase after activation, indicating a large defect density [48]. Typically the Raman spectra of functionalized materials have significantly enhanced D-bands and broadened G-bands [49]. This indicates that physical activation causes the formation of particles with large structural defects.

Taking into account the potential adsorption properties of the obtained biocarbons, the chemical nature of the surface was studied. It was found that the obtained biocarbons are basic (pH~10.86–11.62, Table 3), which at the same time suggests the predominance of surface functional groups of a basic nature. In the research of [50] it was shown that with the increasing temperature and pyrolysis/activation time, the alkalinity of a biocarbon increases and the acidity decreases due to the degradation of acidic surface functional groups. Moreover, CO_2_-activated biocarbons are more alkaline in nature due to the possibility of the formation of metal oxides with the inorganic components of the biocarbon [51]. The data contained in Table 3 show that the obtained biocarbons are characterized by a significant predominance of alkaline functionalities. The most effective was the activation of the biocarbon with the use of overheated water vapor, resulting in the enrichment of the surface with basic functional groups, the presence of which affects the adsorption capacity of the biocarbon [52].

The presence of surface functionalities was confirmed by the Boehm titration analysis [39,40,41]. The analysis consists of the neutralization of the surface functional groups by a suitable acid or base. It was confirmed that both acidic and basic groups occur on the surface of the studied biocarbons, with a predominance of the latter (from 1.57 to ~2.2 mmol/g, Table 3). This suggests that the studied biocarbons will be good potential adsorbents for the adsorption of anionic substances. The sorption capacity depends also on the size of the specific surface area and porosity. The analysis of structural parameters (Table 1) and the content of surface functional groups (Table 3) indicates that the material with the largest sorption capacity should be biocarbon 800-H_2_O-1.

**Figure 5:**
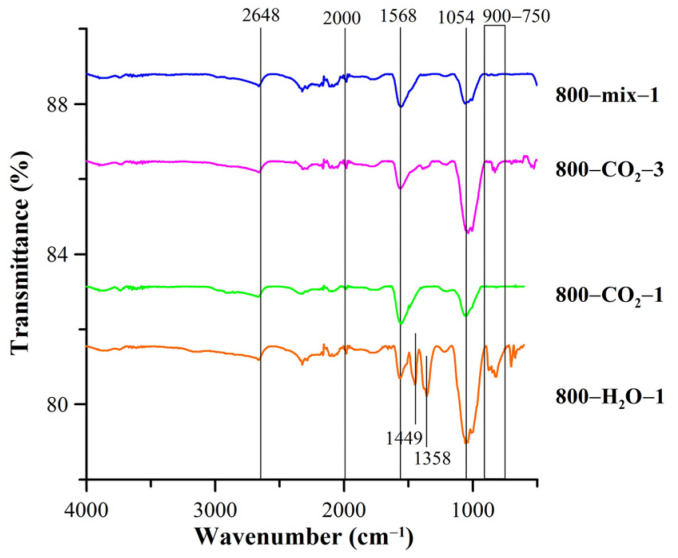
FTIR spectra of the activated biocarbons.

The presence of surface functional groups was also confirmed by the FTIR analysis (Figure 5). The bands of low intensity at ~3700–3500 cm^−1^ correspond to the tensile vibrations of OH groups and may result from the presence of non-associative water molecules since the studies were carried out in an air atmosphere [25]. The band in the range of 2300–2000 cm^−1^ is attributed to C=O stretching aldehydes, ketones, and esters [53]. The bands at 1568 cm^−1^ may be associated with the vibrations of the C=C or C=O groups present in ketones and carboxyl groups [54]. The bands at 1500–1200 cm^−1^, especially in the case of 800-H_2_O-1 and 800-CO_2_-3 biocarbons, are associated with the presence of C=O carbonyl groups [55] and confirm the results obtained using the Boehm method. The wide band in the area of 1568 cm^−1^ results from the C=C vibrations in the aromatic rings. The bands in the area of 900–750 cm^−1^ are associated with the deformation vibrations C-H outside the plane aromatic rings [56].

Activated carbons have important commercial applications for removing organic molecules from air and water. Removal takes place as a result of attractive interactions of the particles with the walls of micropores with dimensions comparable to those of the molecules, among other reasons. The study focused on the adsorption of tetracycline (TC), naproxen (NPX), and methyl orange (MO). Table 4 presents the structures of selected compounds.

The applied adsorbates are characterized by a different structure and particle size, which significantly affects the possibility of their adsorption on the surface of the studied biocarbons. Table 5 shows the total adsorption capacities of biocarbons in relation to impurities.

**Figure 6:**
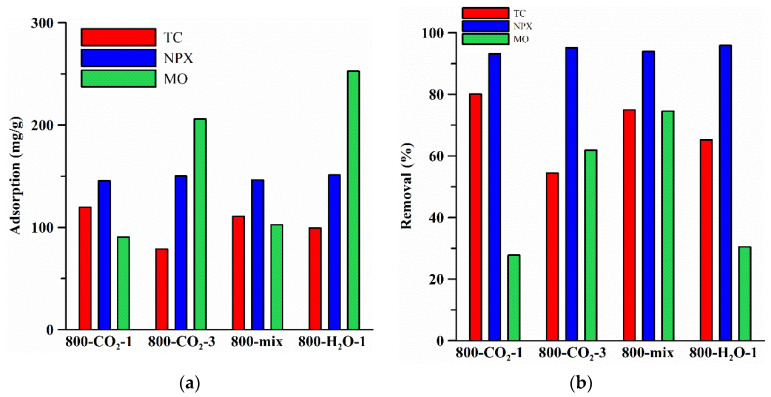
Changes with the adsorption capacities (**a**) and the %R (**b**) for biocarbons.

As is commonly known, the ionic form of tetracycline depends on the pH of the solution [58]. TC has four pK_a_ constant values, indicating that it can exist in different ionic forms. In the presented studies, the pH of the suspension after the TC adsorption process was: 800-H_2_O: 8.75; 800-mix: 8.09; 800-CO_2_-1: 8.01; 800-CO_2_-3: 8.76. At the pH of the solution, >8 TC is mainly in the anionic form and is in the non-dissociated form in small amounts. High pH values of the solutions after contact with the surface of biocarbons (Table 3) indicate that there are positive charges on the surface, favoring the TC adsorption process in the anionic form. The largest sorption capacities (22.87 and 119.79 mg/g, Table 5, Figure 6) were determined for 800-CO_2_-1. Extending CO_2_ activation to 3 h resulted in a reduction in the adsorption capacity. The same trend was observed for the sample obtained in a mixed gas atmosphere and activated by water vapor (Table 5, Figure 6). The alkaline pH of the solutions would suggest a mutual electrostatic attraction between the negatively charged TC molecules and the positively charged surface of the biocarbon. However, at higher pH values, TC adopts an expanded conformation [59], which can affect its interactions with the biocarbon surface. Detailed kinetic studies are needed for a thorough understanding of the mechanism of TC adsorption on biocarbon.

Sodium naproxen takes an anionic form in solutions with pH values higher than its dissociation constant pK_a_ (~4.2), which results in a joint electrostatic attraction between the negatively charged naproxen ions and the positively charged surface of the biocarbon (pH = 10.86–11.62, Table 3) [60]. The pH of the adsorption solutions ranged from 8.39 (800-CO_2_-1) to 9.19 (800-H_2_O-1). This suggests favorable adsorption on the biocarbons. Table 5 presents the detailed data. The adsorption capacities for all biocarbons are similar and range from 22.56 to 25.05 mg/g and 145.53 to 151.28 mg/g for the solutions with C_0_ of 50 and 300 mg/L, respectively (Table 5, Figure 6). The results indicate that NPX adsorbed best on 800-H_2_O-1 (25.06 and 151.28 mg/g, Table 5, Figure 6), which could be due to it having the largest specific surface area and the highest pH value of the biocarbons. In addition, the adsorption pH of the NPX solution filtrate was the highest, indicating better adsorption for the de-protonated NPX form. The developed specific surface area and the content of surface functional groups could affect the number of active sites for adsorption. Increasing the concentration of the adsorbate solution from 50 to 300 mg/L promoted its adsorption on the biocarbon by increasing the number of NPX molecules available for interactions with the adsorbent and facilitating their transport to the active sites due to the small particle size (Table 4) [61].

The adsorption of MO molecules depends on the pH of the solution due to the dissociation of MO and the detachment of Na^+^ ion at pH ≥ 7 as well as the transition to the anionic form [62]. In the case of the tested materials, the alkaline nature of the surface predominates, which can indicate conditions conducive to adsorption of the dye. The adsorption capacities ranged from 90.56 to 252.67 mg/g (Table 5, Figure 6). The extension of the activation time (800-CO_2_-3) and the use of superheated steam (800-H_2_O-1) modification resulted in a significant increase in the sorption capacity of biocarbons (to 206.01 and 252.67 mg/g, respectively, Table 5). This may result from them having the largest specific surface area (Table 1) and the large content of surface functional groups (Table 3).

Figure 6 shows the total sorption capacities of TC, NPX, and MO on the biocarbons (Figure 6a) and the removal coefficients (%R, Figure 6b). Comparing the adsorption of TC and NPX (C_0_ = 300 mg/L), it can be seen that the adsorption of NPX is more effective due to the much smaller particle size, among others. The high %R values (Figure 6b, Table 6) confirm the effective adsorption of the tested pollutants. A decrease in the %R value was observed with an increase in TC and NPX concentrations. Such an effect may result from blocking the places accessible to adsorption on the surface of materials [63]. The highest %R values were determined for NPX (Table 6, Figure 6b).

Table 7 presents the comparison of the total adsorption capacities of biocarbons and activated carbons used for the adsorption of TC, NPX, and MO. The analysis of the presented results indicates that the ability of biocarbons to remove impurities depends on the origin of the biomass, the pyrolysis/activation conditions, as well as the nature and starting concentration of the adsorbate. It can be seen that adsorption on chestnut biocarbons is competitive compared to the materials obtained from other types of biomass and using different activation methods (Table 7). The highest adsorption capacity against TC was shown by the biocarbon from apricot nut shells [64] (q_m_~308 mg/g) activated using H_3_PO_4_. Considering CO_2_ as an activating agent, 800-CO_2_-1 biocarbon (q_e_~120 mg/g) exhibits TC adsorption comparable to that obtained from the Durian shell powder [65] (q_m_~126 mg/g). In the case of NPX, 800-H_2_O-1 showed a similar sorption capacity to that of a biocarbon obtained from sewage sludge [66] (q_m_~151 mg/g vs. 123–128 mg/g). It was also very effective in the process of MO adsorption (q_m_~253 mg/g). Greater efficiency of MO adsorption, taking the cited sources into account, was characterized only by KOH chemically activated materials [67,68] (q_m_~264 mg/g and ~680 mg/g). It was observed that the biocarbons described in this paper show good adsorption properties compared to other materials presented in the cited literature (Table 7).

## 4. Conclusions

The organic biomass (ground chestnut seeds) was successfully used to obtain biocarbons by pyrolysis and physical activation using CO_2_ or superheated steam. Obtained were micro/mesoporous materials with a S_BET_ area of ~540–663 m^2^/g, in which micropores constituted about 70%, thus ensuring great efficiency of the adsorption of organic pollutants. The activation method significantly affected the physicochemical properties, surface chemistry, as well as thermostability of biocarbons. The largest thermal stability was shown by the biocarbon obtained using CO_2_ only at the activation stage (800-mix-1), while the largest adsorption capacity was found in the case of the vapor-activated biocarbon (800-H_2_O-1) due to its well-developed surface area and a significant number of surface functional groups. The studies of total sorption capacities have shown excellent ability to remove organic pollutants, including tetracycline (TC) (q_e_~22–120 mg/g), naproxen (NPX) (q_e_~22–151 mg/g), and methyl orange (MO) (q_e_~90–252 mg/g). High removal rates (%R) confirmed the large adsorption efficiency of biocarbons.

It was shown that the physical activation procedure using mixed gases (N_2_/CO_2_/N_2_) is the most optimal for obtaining an effective material (800-mix-1) for adsorption applications. The research results have important implications for environmental protection and sustainable development. They indicate the high efficiency of chestnut seed biocarbons in adsorption processes, which is important in many areas of industry. These materials are relatively cheap and do not produce significant amounts of by-products which are expensive to dispose of. Future research should focus on optimizing the activation parameters and assessing the long-term adsorption performance of these materials.

## Figures and Tables

**Table 1:** Structural characteristics of the obtained biocarbons.

Biocarbon	S_BET_ [m^2^/g]	S_micro_ [m^2^/g]	S_meso_ [m^2^/g]	V_total_ [cm^3^/g]	R_dom_ [nm]	R_av_ [nm]
800-CO_2_-1	534.6	448.7	85.8	1.393 ± 0.0042	0.94	1.2
800-CO_2_-3	569.5	463.0	106.4	1.590 ± 0.0054	0.99	1.3
800-mix-1	644.0	526.7	117.2	1.976 ± 0.0039	0.96	1.3
800-H_2_O-1	663.1	547.5	115.6	1.125 ± 0.1134	0.95	1.3
**Biocarbon**	**V_p_ [cm^3^/g]**	**V_micro_ [cm^3^/g]**	**V_meso_ [cm^3^/g]**	**ρ [g/cm^3^]**	**w**	**PY [%]**
800-CO_2_-1	0.251	0.182	0.067	0.238 ± 0.0029	0.31	18.5
800-CO_2_-3	0.280	0.189	0.089	0.273 ± 0.0137	0.25	8.7
800-mix-1	0.310	0.212	0.096	0.246 ± 0.0051	0.29	20.2
800-H_2_O-1	0.315	0.219	0.094	0.241 ± 0.0123	0.25	9.6

where S_BET_ is the specific surface area; S_micro_ is the micropores area; S_meso_ is the mesopores area; V_p_ is the total sorption pore volume; V_micro_ is the micropores volume; V_meso_ is the mesopores volume; V_total_ is the total pore volume; R_dom_ is the dominant radius; R_av_ is the mean pore radius; ρ is the bulk density; w is the deviation from the assumed (slit-shaped) pores model; PY is the pyrolysis yield.

**Table 2:** Results of thermal analysis of initial biomass and activated biocarbons.

TG	DTA_max_	%A	%VC	%FC	C_thermo_	T50	R50
INI	330; 473	1.93	75.9	22.1	0.226	343	0.387
800-CO_2_-1	374; 830	5.86	25.9	68.2	0.724	530	0.599
800-CO_2_-3	370; 660	23.5	23.9	52.6	0.687	484	0.546
800-mix-1	376; 820	3.54	17.0	79.4	0.823	543	0.612
800-H_2_O-1	334; 700	18.33	19.2	62.4	0.765	487	0.550

where DTA_max_ is the temperature at which combustion is most effective [°C]; %A is the ash content [%]; %VC is the volatile compound content [%]; %FC is the solid carbon content [%]; C_thermo_ is the thermostability coefficient, C_thermo_ = %FC/(%FC + %VC); T50 is the decomposition temperature of 50% carbon material (after moisture and ash correction), R50 is the recalcitrance index, R50_x_ = T50_x_/T50_graphite_; T50_x_ and T50_graphite_ (886 °C) [43] are the temperatures corresponding to 50% oxidation/volatilization of biocarbon x and graphite, respectively.

**Table 3:** Chemical nature of the surface of the studied biocarbons.

Biocarbon	Acidic Groups [mmol/g]	Basic Groups [mmol/g]	pH
800-CO_2_-1	0.07 ± 0.0202	1.62 ± 0.0116	10.86 ± 0.0208
800-CO_2_-3	0.13 ± 0.0059	2.08 ± 0.0060	11.38 ± 0.0265
800-mix-1	0.01 ± 0.0041	1.57 ± 0.0083	11.23 ± 0.0513
800-H_2_O-1	0.01 ± 0.0028	2.20 ± 0.0406	11.62 ± 0.0153

**Table 4:** Structures of selected adsorbates [57].

Name	Structure	Molecular Weight [g/mol]	Molecular Formula	Topological Polar Surface Area, nm^2^
Tetracycline hydrochloride (TC)	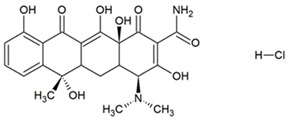	480.9	C_22_H_25_ClN_2_O_8_	1.82
Sodium naproxen (NPX)	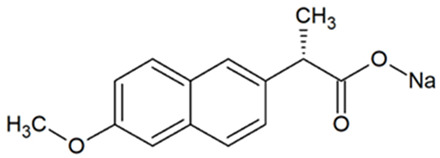	252.2	C_14_H_13_NaO_3_	0.494
Methyl orange (MO)	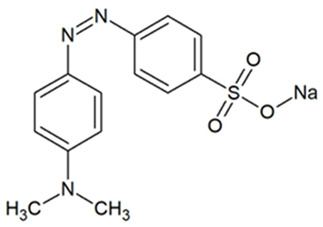	327.3	C_14_H_14_N_3_NaO_3_S	0.935

**Table 5:** Adsorption total capacities of biocarbons as regards TC, NPX, and MO.

Biocarbon	TC Adsorption mg/g		NPX Adsorption mg/g		MO Adsorption mg/g
	C_0_ = 50 mg/L	C_0_ = 300 mg/L	C_0_ = 50 mg/L	C_0_ = 300 mg/L	C_0_ = 700 mg/L
800-CO_2_-1	22.87 ± 0.0479	119.79 ± 1.8811	22.56 ± 0.4493	145.53 ± 2.9525	90.56 ± 2.0878
800-CO_2_-3	22.10 ± 1.7532	97.28 ± 0.0939	24.89 ± 0.0809	150.18 ± 0.7860	206.01 ± 8.9964
800-mix-1	22.29 ± 0.1679	115.77 ± 1.2068	23.73 ± 0.3742	146.30 ± 0.2519	102.58 ± 6.5172
800-H_2_O-1	22.15 ± 0.0672	99.43 ± 1.3557	25.06 ± 0.1611	151.28 ± 2.0165	252.67 ± 1.5711

**Table 6:** Biocarbons removal efficiency (%R).

Biocarbon	TC %R		NPX %R		MO %R
	C_0_ = 50 mg/L	C_0_ = 300 mg/L	C_0_ = 50 mg/L	C_0_ = 300 mg/L	C_0_ = 700 mg/L
800-CO_2_-1	91.80 ± 1.0236	80.08 ± 0.7880	88.03 ± 1.7534	93.13 ± 0.1096	27.82 ± 0.6334
800-CO_2_-3	96.26 ± 0.3697	67.44 ± 0.0651	98.08 ± 0.3187	95.15 ± 0.3889	61.90 ± 2.9222
800-mix-1	94.03 ± 1.1794	76.46 ± 0.7971	96.92 ± 1.6370	93.96 ± 0.6321	30.48 ± 1.9633
800-H_2_O-1	92.89 ± 0.4488	69.90 ± 1.1856	99.24 ± 0.0868	95.91 ± 0.5389	74.56 ± 0.3443

**Table 7:** Comparison of adsorption capacities of carbon materials.

Precursor	Preparation (Pyrolysis Temp.)	Activation	SSA (m^2^/g)	C_0_ (mg/L)	q_m_ TC (mg/g)	q_m_ NPX (mg/g)	q_m_ MO (mg/g)	Ref.
Cow manure	Pyrolysis, 300 °C	-	1.55	10–80	26.73	-	-	[69]
Cow manure	Pyrolysis, 500 °C	-	1.77	10–80	15.06	-	-	[69]
Cow manure	Pyrolysis, 700 °C	-	31.23	10–80	22.55	-	-	[69]
Apricot nut shells	Pyrolysis, 400 °C	H_3_PO_4_ imp.	307.6	100–200	308.33	-	-	[64]
Debarked loblolly pine chips	Pyrolysis, 300 °C	-	1.4	10–100	29.42	-	-	[70]
Durian shell powder	Pyrolysis, 550 °C	CO_2_	917	250	126.08	-	-	[65]
Grape marc	Pyrolysis, 300 °C	HCl	4.25	10	6.56	-	-	[71]
Grape marc	Pyrolysis, 500 °C	HCl	25.94	10	14.01	-	-	[71]
Grape marc	Pyrolysis, 700 °C	HCl	44.23	10	17.88	-	-	[71]
Chestnut seeds	Pyrolysis, 800 °C	CO_2_	534.6	300	119.79	-	-	This work
Sewage sludge	Pyrolysis, 500–700 °C	CO_2_	-	-	-	123–128	-	[66]
Peanut shells	Pyrolysis, 800 °C	air atm.	571	5–1000	-	105	-	[72]
Peanut shells	Pyrolysis, 800 °C	air atm. repeated 2 times	596	5–1000	-	215	-	[72]
Bamboo	Pyrolysis, 800–1000 °C	-	459.8	10–1000	-	56.49	-	[72]
Torrefied loblolly pine chips	Pyrolysis, 300 °C	-	1360	20 (μM)	-	290	-	[73]
Torrefied loblolly pine chips	Pyrolysis, 300 °C	O_2_	1151	20 (μM)	-	228	-	[73]
Walnut shell	Pyrolysis, 700 °C	Pyrolysis, 700 °C	649.9	10–400	-	58.87	-	[74]
Chestnut seeds	Pyrolysis, 800 °C	H_2_O	663.1	300	-	151.28	-	This work
Birchwood pellets	Pyrolysis, 450–500 °C	CO_2_, 900 °C	437.34	500–2500	-	-	153.99	[75]
Chicken manure	Pyrolysis, 600 °C	-	-	40–90	-	-	39.37	[76]
Egyptian doum palm shells	Calcining, 500 °C	NaOH	3.38	25–300	-	-	264.92	[67]
Pomelo peel	Pyrolysis, 450 °C	KOH, 800 °C	1892.1	50–300	-	-	680.3	[68]
Sheep manure	Pyrolysis, 600 °C	HCl	181.76	10–80	-	-	42.51	[77]
Chestnut seeds	Pyrolysis, 800 °C	H_2_O	663.1	700	-	-	252.67	This work

## Data Availability

The original contributions presented in this study are included in the article Further inquiries can be directed to the corresponding author(s).

## References

[1] Pallarés J., González-Cencerrado A., Arauzo I. (2018). Production and Characterization of Activated Carbon from Barley Straw by Physical Activation with Carbon Dioxide and Steam. Biomass Bioenergy.

[2] Yahya M.A., Al-Qodah Z., Ngah C.W.Z. (2015). Agricultural Bio-Waste Materials as Potential Sustainable Precursors Used for Activated Carbon Production: A Review. Renew. Sustain. Energy Rev..

[3] Dsikowitzky L., Schwarzbauer J. (2014). Industrial Organic Contaminants: Identification, Toxicity and Fate in the Environment. Environ. Chem. Lett..

[4] Tkaczyk A., Mitrowska K., Posyniak A. (2020). Synthetic Organic Dyes as Contaminants of the Aquatic Environment and Their Implications for Ecosystems: A Review. Sci. Total Environ..

[5] Wilkinson J.L., Boxall A.B.A., Kolpin D.W., Leung K.M.Y., Lai R.W.S., Galbán-Malagón C., Adell A.D., Mondon J., Metian M., Marchant R.A. (2022). Pharmaceutical Pollution of the World’s Rivers. Proc. Natl. Acad. Sci. USA.

[6] Ślósarczyk K., Jakóbczyk-Karpierz S., Różkowski J., Witkowski A.J. (2021). Occurrence of Pharmaceuticals and Personal Care Products in the Water Environment of Poland: A Review. Water.

[7] Lee C.S., Robinson J., Chong M.F. (2014). A Review on Application of Flocculants in Wastewater Treatment. Process Saf. Environ. Prot..

[8] Muruganandam L., Saravana Kumar M.P., Jena A., Gulla S., Godhwani B. (2017). Treatment of Waste Water by Coagulation and Flocculation Using Biomaterials. IOP Conference Series: Materials Science and Engineering.

[9] Deng Y., Zhao R. (2015). Advanced Oxidation Processes (AOPs) in Wastewater Treatment. Curr. Pollut. Rep..

[10] Pandis P.K., Kalogirou C., Kanellou E., Vaitsis C., Savvidou M.G., Sourkouni G., Zorpas A.A., Argirusis C. (2022). Key Points of Advanced Oxidation Processes (AOPs) for Wastewater, Organic Pollutants and Pharmaceutical Waste Treatment: A Mini Review. ChemEngineering.

[11] Oller I., Malato S., Sánchez-Pérez J.A. (2011). Combination of Advanced Oxidation Processes and Biological Treatments for Wastewater Decontamination—A Review. Sci. Total Environ..

[12] Kathi S., El Din Mahmoud A. (2024). Trends in Effective Removal of Emerging Contaminants from Wastewater: A Comprehensive Review. Desalination Water Treat..

[13] Karpińska J., Kotowska U. (2019). Removal of Organic Pollution in the Water Environment. Water.

[14] Jedynak K., Charmas B. (2023). Activated Biocarbons Obtained from Lignocellulosic Precursors as Potential Adsorbents of Ammonia. Physicochem. Probl. Miner. Process..

[15] Singh N.B., Nagpal G., Agrawal S. (2018). Rachna Water Purification by Using Adsorbents: A Review. Environ. Technol. Innov..

[16] Ali I., Gupta V.K. (2006). Advances in Water Treatment by Adsorption Technology. Nat. Protoc..

[17] Wiśniewska M., Nowicki P., Gruszczyńska K., Urban T. (2022). Activated Biocarbons Obtained from Post-Fermentation Residue as Potential Adsorbents of Organic Pollutants from the Liquid Phase. Physicochem. Probl. Miner. Process..

[18] Amalina F., Razak A.S.A., Krishnan S., Sulaiman H., Zularisam A.W., Nasrullah M. (2022). Biochar Production Techniques Utilizing Biomass Waste-Derived Materials and Environmental Applications—A Review. J. Hazard. Mater..

[19] Mp D., Misra M., Mohanty A.K. (2023). Recent Advances on Value-Added Biocarbon Preparation by the Pyrolysis of Renewable and Waste Biomass, Their Structure and Properties: A Move toward an Ecofriendly Alternative to Carbon Black. Environ. Sci. Adv..

[20] Li S., Tasnady D. (2023). Biochar for Soil Carbon Sequestration: Current Knowledge, Mechanisms, and Future Perspectives. C.

[21] Skubiszewska-Zięba J., Charmas B., Kołtowski M., Oleszczuk P. (2017). Active Carbons from Waste Biochars: Structural and Thermal Properties. J. Therm. Anal. Calorim..

[22] Álvarez-Torrellas S., Rodríguez A., Ovejero G., García J. (2016). Comparative Adsorption Performance of Ibuprofen and Tetracycline from Aqueous Solution by Carbonaceous Materials. Chem. Eng. J..

[23] Jedynak K., Charmas B. (2024). Adsorption Properties of Biochars Obtained by KOH Activation. Adsorption.

[24] Farma R., Anakis R.P., Apriyani I. (2021). Activated Carbons (AC) Prepared by Direct CO_2_ Activation of Parsea Americana Seeds Biomass for Supercapacitor Electrodes. Journal of Physics: Conference Series.

[25] Sajjadi B., Chen W.-Y., Egiebor N.O. (2019). A Comprehensive Review on Physical Activation of Biochar for Energy and Environmental Applications. Rev. Chem. Eng..

[26] Angın D., Altintig E., Köse T.E. (2013). Influence of Process Parameters on the Surface and Chemical Properties of Activated Carbon Obtained from Biochar by Chemical Activation. Bioresour. Technol..

[27] Panwar N.L., Pawar A. (2022). Influence of Activation Conditions on the Physicochemical Properties of Activated Biochar: A Review. Biomass Convers. Biorefin..

[28] Wigmans T. (1989). Industrial Aspects of Production and Use of Activated Carbons. Carbon.

[29] Charmas B., Zięzio M., Jedynak K. (2023). Assessment of the Porous Structure and Surface Chemistry of Activated Biocarbons Used for Methylene Blue Adsorption. Molecules.

[30] Molina-Sabio M., Gonzalez M.T., Rodriguez-Reinoso F., Sepúlveda-Escribano A. (1996). Effect of Steam and Carbon Dioxide Activation in the Micropore Size Distribution of Activated Carbon. Carbon.

[31] Chang C.-F., Chang C.-Y., Tsai W.-T. (2000). Effects of Burn-off and Activation Temperature on Preparation of Activated Carbon from Corn Cob Agrowaste by CO2 and Steam. J. Colloid Interface Sci..

[32] Walker P.L. (1996). Production of Activated Carbons: Use of CO_2_ versus H_2_O as Activating Agent. Carbon.

[33] Marsh H., Rodríguez-Reinoso F. (2006). Activation Processes (Chemical). Activated Carbon.

[34] Januszewicz K., Cymann-Sachajdak A., Kazimierski P., Klein M., Łuczak J., Wilamowska-Zawłocka M. (2020). Chestnut-Derived Activated Carbon as a Prospective Material for Energy Storage. Materials.

[35] Durak H., Genel S. (2024). Investigation of the Effect of Metal Powder and Metal-Supported MCM-41 Catalysts on the Pyrolysis of Horse Chestnut Fruits and Shells (*Aesculus hippocastanum* L.). Biomass Conv. Biorefin..

[36] Haul R.S.J. (1982). Gregg, K.S.W. Sing: Adsorption, Surface Area and Porosity. 2. Auflage, Academic Press, London 1982. 303 Seiten. Berichte Bunsenges. Phys. Chem..

[37] Gun’ko V.M. (2014). Composite Materials: Textural Characteristics. Appl. Surf. Sci..

[38] Gun’ko V.M., Mikhalovsky S.V. (2004). Evaluation of Slitlike Porosity of Carbon Adsorbents. Carbon.

[39] Boehm H.P. (2002). Surface Oxides on Carbon and Their Analysis: A Critical Assessment. Carbon.

[40] Charmas B., Wawrzaszek B., Jedynak K. (2024). Effect of Pyrolysis Temperature and Hydrothermal Activation on Structure, Physicochemical, Thermal and Dye Adsorption Characteristics of the Biocarbons. ChemPhysChem.

[41] Goertzen S.L., Thériault K.D., Oickle A.M., Tarasuk A.C., Andreas H.A. (2010). Standardization of the Boehm Titration. Part I. CO2 Expulsion and Endpoint Determination. Carbon.

[42] Calvelo Pereira R., Kaal J., Camps Arbestain M., Pardo Lorenzo R., Aitkenhead W., Hedley M., Macías F., Hindmarsh J., Maciá-Agulló J.A. (2011). Contribution to Characterisation of Biochar to Estimate the Labile Fraction of Carbon. Org. Geochem..

[43] Harvey O.R., Kuo L.-J., Zimmerman A.R., Louchouarn P., Amonette J.E., Herbert B.E. (2012). An Index-Based Approach to Assessing Recalcitrance and Soil Carbon Sequestration Potential of Engineered Black Carbons (Biochars). Environ. Sci. Technol..

[44] Nowicki P. (2016). The Effect of Mineral Matter on the Physicochemical and Sorption Properties of Brown Coal-Based Activated Carbons. Adsorption.

[45] Naima A., Ammar F., Abdelkader O., Rachid C., Lynda H., Syafiuddin A., Boopathy R. (2022). Development of a Novel and Efficient Biochar Produced from Pepper Stem for Effective Ibuprofen Removal. Bioresour. Technol..

[46] Thommes M., Kaneko K., Neimark A.V., Olivier J.P., Rodriguez-Reinoso F., Rouquerol J., Sing K.S.W. (2015). Physisorption of Gases, with Special Reference to the Evaluation of Surface Area and Pore Size Distribution (IUPAC Technical Report). Pure Appl. Chem..

[47] Ferrari A.C., Robertson J. (2004). Raman Spectroscopy of Amorphous, Nanostructured, Diamond–like Carbon, and Nanodiamond. Philos. Trans. Math. Phys. Eng. Sci..

[48] Martins Ferreira E.H., Moutinho M.V.O., Stavale F., Lucchese M.M., Capaz R.B., Achete C.A., Jorio A. (2010). Evolution of the Raman Spectra from Single-, Few-, and Many-Layer Graphene with Increasing Disorder. Phys. Rev. B.

[49] Li Z., Deng L., Kinloch I.A., Young R.J. (2023). Raman Spectroscopy of Carbon Materials and Their Composites: Graphene, Nanotubes and Fibres. Prog. Mater. Sci..

[50] Gao N., Li J., Qi B., Li A., Duan Y., Wang Z. (2014). Thermal Analysis and Products Distribution of Dried Sewage Sludge Pyrolysis. J. Anal. Appl. Pyrolysis.

[51] Bagreev A., Bandosz T.J., Locke D.C. (2001). Pore Structure and Surface Chemistry of Adsorbents Obtained by Pyrolysis of Sewage Sludge-Derived Fertilizer. Carbon.

[52] Méndez A., Gascó G., Freitas M.M.A., Siebielec G., Stuczynski T., Figueiredo J.L. (2005). Preparation of Carbon-Based Adsorbents from Pyrolysis and Air Activation of Sewage Sludges. Chem. Eng. J..

[53] Bouchelta C., Medjram M.S., Bertrand O., Bellat J.-P. (2008). Preparation and Characterization of Activated Carbon from Date Stones by Physical Activation with Steam. J. Anal. Appl. Pyrolysis.

[54] Jing F., Pan M., Chen J. (2018). Kinetic and Isothermal Adsorption-Desorption of PAEs on Biochars: Effect of Biomass Feedstock, Pyrolysis Temperature, and Mechanism Implication of Desorption Hysteresis. Environ Sci Pollut Res.

[55] Figueiredo J.L., Pereira M.F.R., Freitas M.M.A., Órfão J.J.M. (1999). Modification of the Surface Chemistry of Activated Carbons. Carbon.

[56] Yusof J., Salleh A., Rashid S.A., Ismail I., Adam S.N. (2014). Characterisation of Carbon Particles (CPs) Derived from Dry Milled Kenaf Biochar. J. Eng. Sci. Technol..

[57] National Library of Medicine. https://pubchem.ncbi.nlm.nih.gov.

[58] Caminati G., Puggelli M. (2011). Europium in Phospholipid Nanoscaffolds for the Photophysical Detection of Antibiotic Traces in Solution. Europium: Compounds, Production and Applications.

[59] Oueslati W. (2019). Effect of Soil Solution pH during the Tetracycline Intercalation on the Structural Properties of a Dioctahedral Smectite: Microstructural Analysis. J. Nanomater..

[60] Tomul F., Arslan Y., Kabak B., Trak D., Kendüzler E., Lima E.C., Tran H.N. (2020). Peanut Shells-Derived Biochars Prepared from Different Carbonization Processes: Comparison of Characterization and Mechanism of Naproxen Adsorption in Water. Sci. Total Environ..

[61] Pap S., Taggart M.A., Shearer L., Li Y., Radovic S., Turk Sekulic M. (2021). Removal Behaviour of NSAIDs from Wastewater Using a P-Functionalised Microporous Carbon. Chemosphere.

[62] Chaukura N., Murimba E.C., Gwenzi W. (2017). Synthesis, Characterisation and Methyl Orange Adsorption Capacity of Ferric Oxide–Biochar Nano-Composites Derived from Pulp and Paper Sludge. Appl. Water Sci..

[63] Diaz-Uribe C., Ortiz J., Duran F., Vallejo W., Fals J. (2023). Methyl Orange Adsorption on Biochar Obtained from Prosopis Juliflora Waste: Thermodynamic and Kinetic Study. ChemEngineering.

[64] Marzbali M.H., Esmaieli M., Abolghasemi H., Marzbali M.H. (2016). Tetracycline Adsorption by H3PO4-Activated Carbon Produced from Apricot Nut Shells: A Batch Study. Process Saf. Environ. Prot..

[65] Yazidi A., Atrous M., Edi Soetaredjo F., Sellaoui L., Ismadji S., Erto A., Bonilla-Petriciolet A., Luiz Dotto G., Ben Lamine A. (2020). Adsorption of Amoxicillin and Tetracycline on Activated Carbon Prepared from Durian Shell in Single and Binary Systems: Experimental Study and Modeling Analysis. Chem. Eng. J..

[66] Czech B., Kończak M., Rakowska M., Oleszczuk P. (2021). Engineered Biochars from Organic Wastes for the Adsorption of Diclofenac, Naproxen and Triclosan from Water Systems. J. Clean. Prod..

[67] Tcheka C., Conradie M.M., Assinale V.A., Conradie J. (2024). Mesoporous Biochar Derived from Egyptian Doum Palm (*Hyphaene thebaica*) Shells as Low-Cost and Biodegradable Adsorbent for the Removal of Methyl Orange Dye: Characterization, Kinetic and Adsorption Mechanism. Chem. Phys. Impact.

[68] Li H., Sun Z., Zhang L., Tian Y., Cui G., Yan S. (2016). A Cost-Effective Porous Carbon Derived from Pomelo Peel for the Removal of Methyl Orange from Aqueous Solution. Colloids Surf. A Physicochem. Eng. Asp..

[69] Zhang P., Li Y., Cao Y., Han L. (2019). Characteristics of Tetracycline Adsorption by Cow Manure Biochar Prepared at Different Pyrolysis Temperatures. Bioresour. Technol..

[70] Jang H.M., Yoo S., Choi Y.-K., Park S., Kan E. (2018). Adsorption Isotherm, Kinetic Modeling and Mechanism of Tetracycline on Pinus Taeda-Derived Activated Biochar. Bioresour. Technol..

[71] Sağlam S., Türk F.N., Arslanoğlu H. (2024). Tetracycline (TC) Removal from Wastewater with Activated Carbon (AC) Obtained from Waste Grape Marc: Activated Carbon Characterization and Adsorption Mechanism. Environ. Sci. Pollut. Res..

[72] Pham T.D., Nguyen D.T., Nguyen H.L., Nguyen M.Q., Tran T.M., Nguyen M.V., Nguyen T.L., Ngo T.M.V., Namakamura K., Tsubota T. (2025). Adsorption Characteristics of Ciprofloxacin and Naproxen from Aqueous Solution Using Bamboo Biochar. Biomass Conv. Biorefin..

[73] Jung C., Boateng L.K., Flora J.R.V., Oh J., Braswell M.C., Son A., Yoon Y. (2015). Competitive Adsorption of Selected Non-Steroidal Anti-Inflammatory Drugs on Activated Biochars: Experimental and Molecular Modeling Study. Chem. Eng. J..

[74] Şensoy R., Kabak B., Kendüzler E. (2024). Kinetic and Isothermal Studies of Naproxen Adsorption from Aqueous Solutions Using Walnut Shell Biochar. React. Kinet. Mech. Catal..

[75] Lee H., Fiore S., Berruti F. (2024). Adsorption of Methyl Orange and Methylene Blue on Activated Biocarbon Derived from Birchwood Pellets. Biomass Bioenergy.

[76] Yu J., Zhang X., Wang D., Li P. (2018). Adsorption of Methyl Orange Dye onto Biochar Adsorbent Prepared from Chicken Manure. Water Sci. Technol..

[77] Lu Y., Chen J., Bai Y., Gao J., Peng M. (2019). Adsorption Properties of Methyl Orange in Waterby Sheep Manure Biochar. Pol. J. Environ. Stud..

